# Determination of Acquisition Frequency for Intrafractional Motion of Pancreas in CyberKnife Radiotherapy

**DOI:** 10.1155/2014/408019

**Published:** 2014-05-13

**Authors:** Huailing Zhang, Guoru Zhao, David Djajaputra, Yaoqin Xie

**Affiliations:** ^1^School of Information Engineering, Guangdong Medical College, Dongguan, Guangdong 523808, China; ^2^Key Laboratory for Health Informatics, Shenzhen Institutes of Advanced Technology, Chinese Academy of Sciences, 1068 Xueyuan Avenue, Shenzhen, Guangdong 518055, China; ^3^Frederick Memorial Hospital, MD 21701, USA; ^4^Department of Radiation Oncology, Stanford University School of Medicine, Stanford, CA 94305, USA

## Abstract

*Purpose.* To report the characteristics of pancreas motion as tracked using implanted fiducials during radiotherapy treatments with CyberKnife. * Methods and Materials*. Twenty-nine patients with pancreas cancer treated using CyberKnife system were retrospectively selected for this study. During the treatment, the deviation is examined every 3-4 nodes (~45 s interval) and compensated by the robot. The pancreas displacement calculated from X-ray images acquired within the time interval between two consecutive couch motions constitute a data set. * Results*. A total of 498 data sets and 4302 time stamps of X-ray images were analyzed in this study. The average duration for each data set is 634 s. The location of the pancreas becomes more dispersed as the time elapses. The acquisition frequency depends on the prespecified movement distance threshold of pancreas. If the threshold between two consecutive images is 1 mm, the acquisition frequency should be less than 30 s, while if the threshold is 2 mm, the acquisition frequency can be around 1 min. * Conclusions*. The pancreas target moves significantly and unpredictably during treatment. Effective means of compensating the intrafractional movement is critical to ensure adequate dose coverage of the tumor target.

## 1. Introduction

Pancreatic cancer remains one of the most formidable challenges in oncology [1]. In 2013, the American Cancer Society estimated that 45,220 new cases of pancreatic cancer in the United States will develop and 38,460 deaths from the disease will result [2].

The role of radiotherapy (RT) for pancreas cancer remains hotly debated. Randomized prospective data using modern RT techniques and dosing are needed. In locally advanced pancreas cancer, recent evidence using modern RT techniques and dosing suggests a continued role for RT [3].

With the technological advancements in image guidance and dose delivery, several advanced radiotherapy technologies, such as stereotactic body radiotherapy (SBRT) and stereotactic radio surgery (SRS), are being widely used for cancer treatment as a noninvasive alternative to surgery. These technologies are characterized by the delivery of a high radiation dose to a small volume in a short time interval with high accuracy as well as conformality [4]. Different from the conventional radiotherapy, radiation dose is delivered in fewer fractions and higher fractional dose in SBRT [5]. For example, SBRT delivers 1 to 5 high-dose radiation fractions, as opposed to conventional RT which delivers 25 to 28 low-dose fractions. The rationale behind conventional fraction RT is that delivering a lower dose of radiation per day minimizes damage to normal tissues. Therefore, substantial amounts of normal tissue can be included in the radiation field [3]. To deliver ablative doses to tumors with limited normal tissue toxicity, these new techniques require high accuracy in treatment setup which requires taking tumor motion into account [5].

These techniques are being increasingly applied to pancreatic cancer, in part due to patient demand and marketing [6]. A phase II trial of SBRT by Hoyer et al. used 30 Gy in 3 fractions. This study demonstrated a local control rate of 57% but unacceptable small bowel toxicity, with 18% of patients experiencing severe gastrointestinal mucositis/ulceration and 4.5% experiencing gastric perforation [7]. In our institution, CyberKnife system was capable of delivering therapeutic radiation with minimal toxicity to tumors in patients with locally advanced pancreatic cancer [8, 9].

The pancreas presents unique difficulties with regard to performing these new technologies. While it is not itself prone to damage from radiation, the pancreas is closely applied to the curve of the duodenum. The duodenum is the primary dose-limiting normal tissue. Delivery of even moderate doses of radiation (more than 50 Gy in 1.8–2 Gy/day fractions) to small bowel is associated with a high risk of late stenosis, ulceration, bleeding, and perforation. The risk of late bowel complications is substantially heightened by the use of large fraction sizes. Tumors of the pancreas also move with respiration and with peristalsis and are relatively difficult to visualize on the computed-tomography scans typically used for treatment planning [6].

To compensate for unquantified geometric uncertainties in target volume position, generic margins are commonly applied to the clinical target volume (CTV) to form a planning target volume (PTV). Such a margin estimate may or may not encompass the “current” extent of motion exhibited by the pancreas. Previous studies have provided some information regarding pancreatic/tumor movement [10–22]. A number of techniques, such as real-time tracking, have been developed for measuring set-up variations and internal organ motions for individual patients from day-to-day and during a fractional treatment [3, 23].

In our institute, by frequent tracking external markers and stereoscopic X-ray imaging of implanted fiducials, the system provides an effective way to monitor the position of the pancreas target during a treatment and adaptively adjust the radiation beam [24–26]. The system correlates and records the position of the center of the mass (COM) of the implanted fiducials as estimated from each pair of stereoscopic images during treatment, thus providing a valuable set of data for us to better understand the intrafractional movement of the pancreas. In this study, we analyze 4302 time stamps recorded by CyberKnife for 29 pancreas patients. The study sheds useful insight into the feature of intrafractional pancreas motion and recommends appropriate imaging frequency to ensure adequate dose coverage of the tumor target.

## 2. Methods and Materials

### 2.1. Ethics Approval

In this study we analysed 29 pancreas cancer patients' data in Stanford Cancer Center, which was approved by the Institutional Review Boards of Stanford University. Written informed consent was obtained for all participants. These data could only be accessed by the physicians and researchers in the center, to ensure participant confidentiality.

### 2.2. Setting and Study Design

Accuray CyberKnife can track using 4 modes: 6D skull tracking for brain, X-sight tracking for spine, fiducial tracking for extracranial (such as prostate), and Synchrony to account for respiratory motion (such as lung and pancreas). X-sight uses the bony anatomy of the spine to automatically locate and track tumors. Synchrony continuously synchronizes beam delivery with the motion of the target resulting from respiration, without the need to interrupt the treatment or move the patient.

The patient setup and treatment delivery process in CyberKnife is as follows [26]. First, orthogonal X-ray images are acquired before treatment. The system determines the absolute position of the target volume via image-to-DRR (digital radiographic reconstruction) registration. The 3D translation of the target from the planned position is calculated. The deviation is corrected by manually moving the treatment couch. The treatment starts if the computed shift is less than a preset threshold, 10 mm in general. During the beam delivery X-ray images are acquired every 40 seconds. The shift of tumor motion is monitored by using image registration process of the treatment radiographs to DRR constructed from the planning CT. If the calculated shift is more than the given threshold, the treatment will be paused and the manual couch movement is required until the shift is below the limit. After patient treatment, a log-file containing COM displacements of the fiducials in anterior/posterior (AP), left/right (LR), and superior/inferior (SI) directions is saved in the CyberKnife control computer and can be readily used for the analysis of organ motion during the beam delivery process.

Totally 29 pancreas cancer patients data are used for the study. The pancreas movement is defined as the displacement of COM of the fiducials from the planned position. One fraction can have more than one data set, because during the treatment, if the pancreas movement is out of the hardware limitation, the patient is manually repositioned.

## 3. Results

### 3.1. Duration of Data Sets

**Figure 1:**
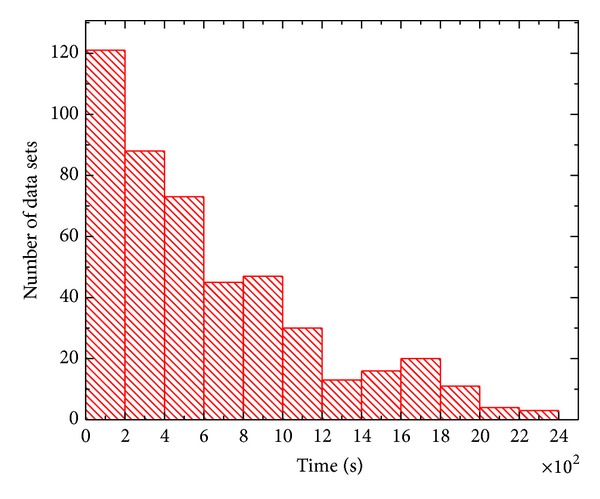
Histogram of the time span of the studied data sets.

For the 29 patients, a total of 498 data sets and 4302 time stamps were recorded. For each patient, the duration of each data set is an indicator of the stability of the tumor target position.  Figure 1 shows the histogram of the duration of the 498 data sets. The* x*-axis and* y*-axis represent the duration and the number of data sets for a given time length, respectively. The time bin size is 200 s and the average duration of the 498 data sets is 634 s. The shorter the duration is, the more “violent” the pancreas moves.

### 3.2. Intrafractional Movement of the Pancreas Target

**Figure 2:**
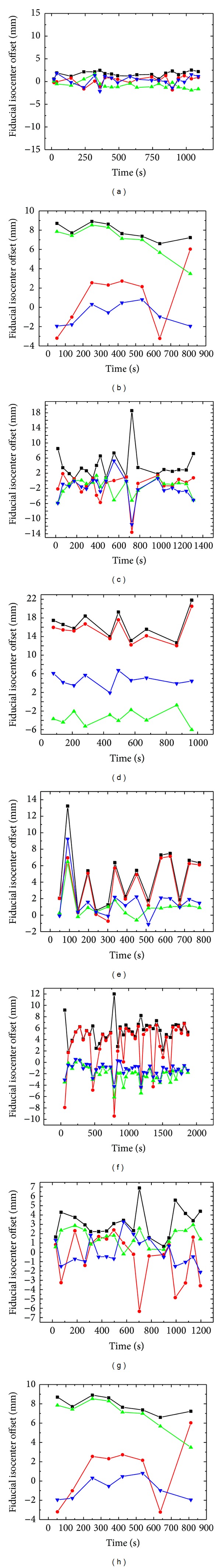
Patterns of pancreas movement: (a) stable positioning at baseline; (b) dropping down; (c) transient excursion; (d) persistent excursion; (e) high-frequency excursions; (f) intermittent vibration; (g) radon movement; (h) and LR moving (red: SI direction; green: LR direction; blue: AP direction; and black: length).

The movement of the COM of the three implanted fiducials is used as a surrogate of pancreas movement. This quantity was recorded continuously over time and analyzed for the 498 data sets (on average, 17 data sets per patient). For convenience, selections of observed motion behaviors are shown in Figure 2. The* x*-axis represents the time stamp, and the* y*-axis is the pancreas displacement. The red, green, and blue lines represent the movement in SI, LR, and AP directions, respectively. The black lines represent the total displacement distance of the pancreas.

The pancreas movement patterns vary from stable positioning at baseline (Figure 2(a)), dropping down (Figure 2(b)), transient excursion (Figure 2(c)), persistent excursion (Figure 2(d)), high-frequency excursions (Figure 2(e)), and intermittent vibration (Figure 2(f)). Some patterns are simply too irregular to categorize into any of the above classes (Figure 2(g)). For most data sets, the major movement is along SI direction, while in some cases the major movement is along LR direction (Figure 2(h)).

It should be noted that, for a given patient, the above classification of the pancreas movement pattern is somewhat arbitrary and it may change from fraction to fraction or even from data set to data set within the same treatment fraction. High-frequency excursion is the most popular movement for pancreas, because of the respiratory movement; that is why the Synchrony respiratory tracking system is used in pancreas positioning.

**Figure 3:**
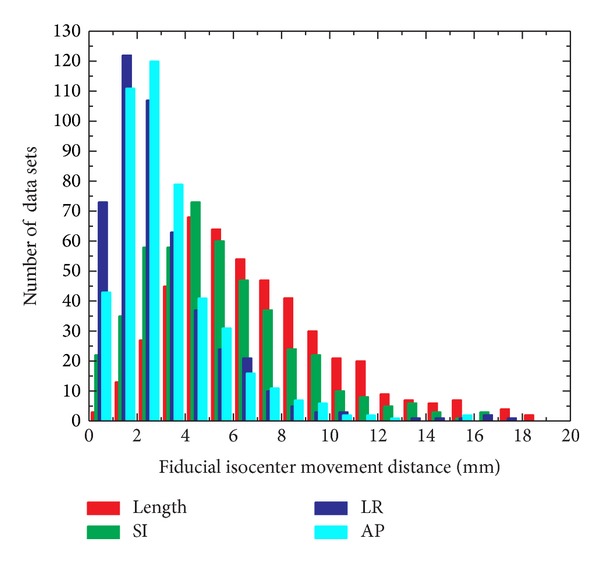
Histogram of the fiducial isocenter movement distance in different directions.

A useful way to present the pancreas motion data is to show the histogram of the fiducial isocenter movement distance in different directions. As seen in Figure 3, the histogram of the deviation distance (length) is similar to the histogram of SI. It is demonstrated that the major movement of pancreas is along SI direction. Generally, the shift distribution in LR direction is similar to that in AP direction, and the amplitude is relatively small compared to the SI direction.

**Figure 4:**
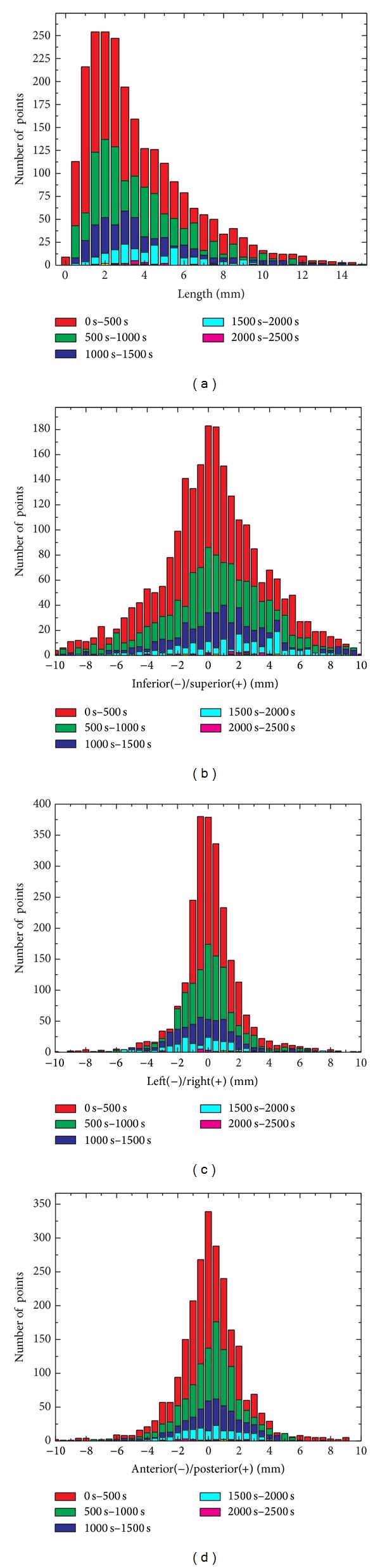
Histogram of the pancreas movement as a function of displacement and time.

An alternative way to present the data of Figure 3 is to illustrate the histogram of the pancreas position as a function of displacement and time as shown in Figure 4. Each color represents a specific time segment. From these plots, it is also clear that the movement in LR and AP direction is small; the major movement is in SI direction.

## 4. Discussions

The effect of RT for pancreas cancer is not clearly defined by the existing data. In both resected and unresected diseases, further study is needed to define optimal radiation dose, field size, and technique and to more closely assess the effect of radiotherapy, not only on survival, but also on local disease control and quality of life [3]. To support increasingly conformal RT techniques that facilitate dose escalation, quantification of pancreatic motion and patient positioning is required.

Indeed, many studies have indicated that the margin used for treatment is either too small for a significant fraction of the pancreas patients, which seriously underdoses the target, or too large for another large fraction of the patients. Solla et al. present that the potential target underdosage may occur due to respiration and small PTV margin [27]. Several studies have shown a survival advantage for higher dose levels [28–33]. In this case, underdosage is not facilitated for overall survival. Other detailed discussions can be seen in [34].

**Figure 5:**
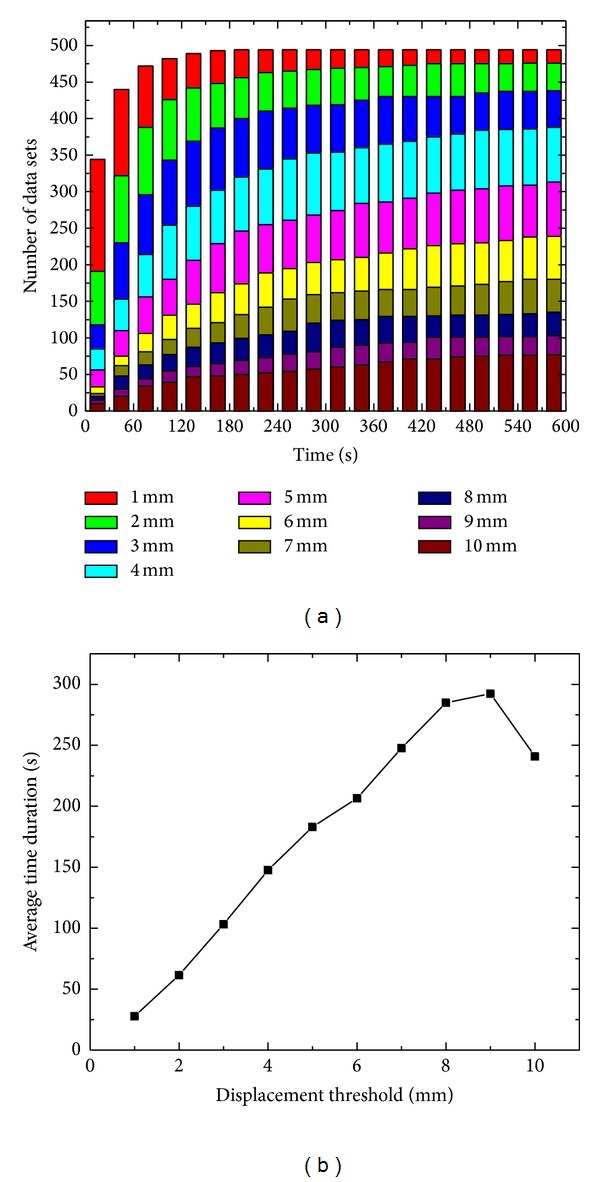
Determination of acquisition time for intrafractional motion of pancreas. (a) Histogram of time cumulative distribution for different movement distances; (b) average time duration against displacement threshold.

A robust strategy in locating the tumor target is necessary to more accurately target the tumor, so that the radiation dose to the pancreas can be escalated without damaging the sensitive structures. Stereoscopic imaging of implanted metallic fiducials is a useful way to locate the pancreas target in nearly real-time fashion. A clinically important question in stereoscopic image-guided pancreas treatment is what the optimal imaging frequency that minimizes the patient exposure while not missing any significant residual movement of the pancreas during the beam-off interval of the imaging X-ray is [35]. In reality, a few factors may influence the selection of the sampling rate of the X-ray imaging, which include the dose rate, patient specific characteristics, and the fractionation scheme. A rule of thumb is that the movement of pancreas within the interval of two consecutive images should be less than a prespecified criterion, say 1 or 2 mm. Due to the randomness of the pancreas movement, this decision can be made based on the average displacement of the pancreas over time.  Figure 5 shows how to determine the acquisition time for intrafractional motion of pancreas.  Figure 5(a) is the histogram of time cumulative distribution for different movement distances. The average time for 1.0 mm motion is 30 s. Using the 498 data sets, we also computed the average time duration for the pancreas against moving distance as shown in Figure 5(b). The data for each movement threshold in Figure 5(a) and the mean sample interval is determined from 0.707 of the total data sets. For instance, the total data sets for 2 mm movement threshold are 478, so that the mean sample interval is about 60 s corresponding to 338 (0.707∗478) data sets. It can be seen that the acquisition frequency depends on the prespecified movement distance threshold of pancreas. If the threshold between two consecutive images is 1 mm, the acquisition frequency should be less than 30 s, while if the threshold is 2 mm, the acquisition frequency can be around 1 min. It is also noticed that the acquisition frequency is proportional to the prespecified movement distance threshold.

There are several drawbacks associated with fiducial based image-guided pancreas radiation therapy. Other than the fact that it involves an invasive procedure of fiducial implantation, the fiducial tracking used in CyberKnife is limited to “rigid” tumors. In reality, tracking the motion of various involved sensitive structures represents the other side of the “coin” and is also of critical importance to the success of image-guided radiation therapy (IGRT) [36, 37]. After all, it is the adjacent sensitive structures that limit the dose deliverable to the target. Therefore, knowing the spatial location and geometric shapes of the sensitive structures is critical for us to customize the dose distribution to maximize the dose to the target while sparing the adjacent sensitive structures. On a fundamental level, the motion of pancreas target is often caused by the motion or physiological change of the sensitive structures.

## 5. Conclusions

Intrafractional organ motion has long been recognized as one of the major limiting factors of pancreas dose escalation in conformal radiation therapy. A detailed knowledge of pancreas motion would help to understand the nature and degree of the adverse influence of the uncertainty and provide guidance in dealing with the issue. In this work, we have studied the intrafractional pancreas motion of 29 pancreas cancer patients. The study emphasizes the importance of real-time imaging during pancreas radiation therapy. Given the magnitude and random nature of pancreas motion as well as the recent technical advancements in various related fields, real-time monitoring of pancreas position to adaptively compensate the motion should be part of future pancreas radiation therapy to ensure adequate dose coverage of the pancreas target while maintaining an adequate sparing of the sensitive structures.

## References

[1] Willett CG, Safran H, Abrams RA, Regine WF, Rich TA (2003). Clinical research in pancreatic cancer: the Radiation Therapy Oncology Group trials. *International Journal of Radiation Oncology, Biology, Physics*.

[2] Siegel R, Naishadlham D, Jemal A (2013). Cancer statistics, 2013. *CA: A Cancer Journal for Clinicians*.

[3] Hazard L (2009). The role of radiation therapy in pancreas cancer. *Gastrointestinal Cancer Research*.

[4] Zhang GG, Yu HHM, Stevens CW (2012). Motion management in stereotactic body radiotherapy. *Journal of Nuclear Medicine & Radiation Therapy*.

[5] Benedict SH, Yenice KM, Followill D (2010). Stereotactic body radiation therapy: the report of AAPM Task Group 101. *Medical Physics*.

[6] Chang BW, Saif MW (2008). Stereotactic body radiation therapy (SBRT) in pancreatic cancer: is it ready for prime time?. *Journal of the Pancreas*.

[7] Hoyer M, Roed H, Hansen AT (2006). Phase II study on stereotactic body radiotherapy of colorectal metastases. *Acta Oncologica*.

[8] Koong AC, Le QT, Ho A (2004). Phase I study of stereotactic radiosurgery in patients with locally advanced pancreatic cancer. *International Journal of Radiation Oncology, Biology, Physics*.

[9] Koong AC, Christofferson E, Le Q-T (2005). Phase II study to assess the efficacy of conventionally fractionated radiotherapy followed by a stereotactic radiosurgery boost in patients with locally advanced pancreatic cancer. *International Journal of Radiation Oncology, Biology, Physics*.

[10] Whitfield G, Jain P, Green M (2012). Quantifying motion for pancreatic radiotherapy margin calculation. *Radiotherapy & Oncology*.

[11] Bussels B, Goethals L, Feron M (2003). Respiration-induced movement of the upper abdominal organs: a pitfall for the three-dimensional conformal radiation treatment of pancreatic cancer. *Radiotherapy & Oncology*.

[12] Bryan PJ, Custar S, Haaga JR, Balsara V (1984). Respiratory movement of the pancreas: an ultrasonic study. *Journal of Ultrasound in Medicine*.

[13] Feng M, Balter JM, Normolle D (2009). Characterization of pancreatic tumor motion using cine MRI: surrogates for tumor position should be used with caution. *International Journal of Radiation Oncology, Biology, Physics*.

[14] Aruga T, Itami J, Aruga M (2000). Target volume definition for upper abdominal irradiation using CT scans obtained during inhale and exhale phases. *International Journal of Radiation Oncology, Biology, Physics*.

[15] Horst E, Micke O, Moustakis C, Schuck A, Schäfer U, Willich NA (2002). Conformal therapy for pancreatic cancer: variation of organ position due to gastrointestinal distention—implications for treatment planning. *Radiology*.

[16] Murphy MJ, Martin D, Whyte R, Hai J, Ozhasoglu C, Le Q-T (2002). The effectiveness of breath-holding to stabilize lung and pancreas tumors during radiosurgery. *International Journal of Radiation Oncology, Biology, Physics*.

[17] Ozhasoglu C, Murphy MJ (2002). Issues in respiratory motion compensation during external-beam radiotherapy. *International Journal of Radiation Oncology, Biology, Physics*.

[18] Gierga DP, Chen GTY, Kung JH, Betke M, Lombardi J, Willett CG (2004). Quantification of respiration-induced abdominal tumor motion and its impact on IMRT dose distributions. *International Journal of Radiation Oncology, Biology, Physics*.

[19] Marchant TE, Amer AM, Moore CJ (2008). Measurement of inter and intra fraction organ motion in radiotherapy using cone beam CT projection images. *Physics in Medicine and Biology*.

[20] Minn AY, Schellenberg D, Maxim P (2009). Pancreatic tumor motion on a single planning 4D-CT does not correlate with intrafraction tumor motion during treatment. *American Journal of Clinical Oncology: Cancer Clinical Trials*.

[21] Jayachandran P, Minn AY, van Dam J, Norton JA, Koong AC, Chang DT (2010). Interfractional uncertainty in the treatment of pancreatic cancer With radiation. *International Journal of Radiation Oncology, Biology, Physics*.

[22] Henry AM, Ryder WDJ, Moore C (2008). Chemoradiotherapy for locally advanced pancreatic cancer: a Radiotherapy Dose Escalation and Organ Motion study. *Clinical Oncology*.

[23] Langen KM, Jones DTL (2001). Organ motion and its management. *International Journal of Radiation Oncology, Biology, Physics*.

[24] Dieterich S, Mould RF (2005). Dynamic tracking of moving tumors in stereotactic radiosurgery. *Robotic Radiosurgery*.

[25] Dieterich S, Suh Y, Urschel HC, Kresl JJ, Luketich JD (2007). Tumor motion ranges due to respiration and respiratory motion characteristics. *Treating Tumors That Move with Respiration*.

[26] Xie Y, Djajaputra D, King CR, Hossain S, Ma L, Xing L (2008). Intrafractional motion of the prostate during hypofractionated radiotherapy. *International Journal of Radiation Oncology, Biology, Physics*.

[27] Solla I, Zucca S, Possanzini M (2013). Free-breathing conformal irradiation of pancreatic cancer. *Journal of Applied Clinical Medical Physics*.

[28] Perez CA, Bauer M, Edelstein S, Gillespie BW, Birch R (1986). Impact of tumor control on survival in carcinoma of the lung treated with irradiation. *International Journal of Radiation Oncology, Biology, Physics*.

[29] Choi NCH, Doucette JA (1981). Improved survival of patients with unresectable non-small-cell bronchogenic carcinoma by an innovated high-dose en-bloc radiotherapeutic approach. *Cancer*.

[30] Martel MK, ten Haken RK, Hazuka MB (1999). Estimation of tumor control probability model parameters from 3-D dose distributions of non-small cell lung cancer patients. *Lung Cancer*.

[31] Okunieff P, Morgan D, Niemierko A, Suit HD (1995). Radiation dose-response of human tumors. *International Journal of Radiation Oncology, Biology, Physics*.

[32] Perez CA, Stanley K, Rubin P (1980). A prospective randomized study of various irradiation doses and fractionation schedules in the treatment of inoperable non-oat-cell carcinoma of the lungs. Preliminary report by the Radiation Therapy Oncology Group. *Cancer*.

[33] Perez CA, Pajak TF, Rubin P (1987). Long-term observations of the patterns of failure in patients with unresectable non-oat cell carcinoma of the lung treated with definitive radiotherapy. Report by the Radiation Therapy Oncology Group. *Cancer*.

[34] Levitt SH, Perez CA, Hui S, Purdy JA (2008). Evolution of computerized radiotherapy in radiation oncology: potential problems and solutions. *International Journal of Radiation Oncology, Biology, Physics*.

[35] Xing L, Thorndyke B, Schreibmann E (2006). Overview of image-guided radiation therapy. *Medical Dosimetry*.

[36] Shinohara ET, Kassaee A, Mitra N (2012). Feasibility of electromagnetic transponder use to monitor inter- and intrafractional motion in locally advanced pancreatic cancer patients. *International Journal of Radiation Oncology, Biology, Physics*.

[37] Xie YQ, Xing L, Gu J, Liu W (2013). Tissue feature-based intra-fractional motion tracking for stereoscopic X-ray image guided radiotherapy. *Physics in Medicine and Biology*.

